# Supplementary material for: Allergic Bronchopulmonary Aspergillosis (ABPA) in the Era of Cystic Fibrosis Transmembrane Conductance Regulator (CFTR) Modulators
Source: J Fungi (Basel). 2024 Sep 18;10(9):656. doi: 10.3390/jof10090656 (PMC11433030; doi:10.3390/jof10090656)
Supplement: Supplementary file 1 [file jof-10-00656-s001.zip › jof-3125295-supplementary.pdf]

Supplement Table S1: CFTR Modulator Clinical Trials

**Drug**	**Study**	**Reference**	**ABPA?**	**Surrogate?**
Ivacaftor	Ramsey et al., 2011	78	No	No
	Quittner et al., 2015	79	No	No
	Solem et al., 2016	80	No	No
	Flume et al., 2018	81	No	No
	Davies et al., 2016	82	No	No
	McGarry et al., 2017	83	No	No
	Edgeworth et al., 2017	84	-	-
	Rosenfeld et al., 2019	86	No	No
	Stallings et al., 2018	87	No	No
	Rosenfeld et al., 2018	85	No	No
Lumacaftor/Ivacaftor	Boyle et al., 2014	88	No	No
	Rowe et al., 2017	89	No	No
	Wainwright et al., 2015	90	No	No
	Elborn et al., 2016	91	No	No
	Flume et al., 2019	92	No	No
	McColley et al., 2019	93	No	No
	Milla et al., 2017	94	No	No
	Konstan et al., 2017	95	No	No
	Taylor-Cousar et al., 2018	96	No	No
	Ratjen et al., 2017	97	No	No
	McNamara et al., 2019	98	No	No
	Graeber et al., 2018	99	No	No
	Pranke et al., 2019	100	No	No
Tezacaftor/Ivacaftor	Taylor-Cousar et al., 2017	101	Yes<br>Placebo	No

			1 case, N=258, 509 total	
	Rowe et al., 2017a	89	No	No
	Donaldson et al., 2018a	102	No	No
	Walker et al., 2019	103	No	No
Elexacaftor/ Tezacaftor/Ivacaftor	Keating et al., 2018	104	No	No
	Middleton et al., 2019	105	No	No
	Heijerman et al., 2019	106	No	No
